# T2 mapping and fat quantification of lumbar paraspinal muscle in ankylosing spondylitis: a case control study

**DOI:** 10.1186/s12891-022-05570-9

**Published:** 2022-06-27

**Authors:** Ruibin Huang, Hongwu Yang, Liujiang Chen, Shuyan Su, Xiaojia Wu, Ruyao Zhuang, Yuan Liu

**Affiliations:** Department of Radiology, First Affiliated Hospital, Shantou University Medical College, Shantou, Guangdong 515041 People’s Republic of China

**Keywords:** Ankylosing spondylitis, Paraspinal muscle, Muscle degeneration, Fat infiltration, T2 mapping, T2 IDEAL

## Abstract

**Background:**

To compare changes in the composition of paraspinal muscles of patients with ankylosing spondylitis (AS) and matched healthy controls using T2 mapping and T2 IDEAL and correlate the quantitative magnetic resonance imaging (qMRI) results with clinical assessments of AS patients.

**Method:**

In total, 37 AS patients and 37 healthy controls were enrolled in the case control study. T2 mapping with and without fat saturation and IDEAL imaging were used to assess the multifidus (MF) and erector spinae (ES) at the levels of L3/L4 and L4/L5 for all subjects. Mean T2_non-fatsat_, T2_fat_, T2_fatsat_, cross-sectional area (CSA), and fat fraction (FF) were compared between AS and healthy controls. Correlations of qMRI results with clinical assessments were analyzed in AS.

**Results:**

Significantly elevated mean T2_non-fatsat_ values and the FF of the MF and ES at both levels were observed in AS and compared to the controls (*p* < 0.05). The mean T2_fatsat_ values of ES and MF were significantly higher only at the level of L3/L4 in AS compared to healthy controls (*p* < 0.05). A loss of muscle CSA compatible with atrophy was present in MF and ES at both levels in AS compared to the controls (*p* < 0.05). Weak to moderate positive correlations were found between FF and age and disease duration in AS (*r* = 0.318–0.415, *p* < 0.05). However, such positive correlation was not observed between FF and disease duration after adjusting for age (*p* > 0.05).

**Conclusions:**

Our findings indicate that using a combination of IDEAL and T2 mapping may provide deeper insights into the pathophysiological degeneration of paraspinal muscles in AS.

**Supplementary Information:**

The online version contains supplementary material available at 10.1186/s12891-022-05570-9.

## Background

Ankylosing spondylitis (AS) is a systemic inflammatory rheumatic disease that can affect the axial and peripheral skeleton, causing characteristic inflammatory lower back pain and structural and functional impairments [1–3]. It has been reported that the paraspinal muscles are affected in AS. Impairment of these muscles has been proposed as a risk factor for radiological progression and has recently received attention as a cause of pain, spinal sagittal balance impairment, spinal deformity, and worsened quality of life [4–11]. As AS treatment advances, core strength and physical exercise have been shown to have a positive effect on disease activity and flexibility in AS [12–14]. Therefore, understanding the pathophysiology and subsequent structural alterations in paraspinal muscle is of high scientific but also of substantial clinical value [8, 10, 15, 16].

A recent study by Ozturk et al. revealed that patients with AS have more fatty degeneration in paraspinal muscles may contribute the severity of pain and disability [14]. In the past, imaging assessment of lumbar paraspinal muscles in AS was mainly using computed tomography and conventional magnetic resonance imaging (MRI) [14, 17–19]. However, these studies are based on qualitative analysis and semi-quantitative measurement of the fat infiltration, which is subjective and may not be as accurate as quantitative measurements, and the standard qualitative analysis may be insufficient to fully characterize degenerative changes of paraspinal muscles in AS [20–22]. Therefore, there is a need for a non-invasive assessment method that can provide quantitative and global information on paraspinal muscle composition in AS.

Currently, novel quantitative (q)MRI techniques, such as T2 relaxation time mapping (T2 mapping) and iterative decomposition of water and fat with echo asymmetry and least square estimation (IDEAL), are increasingly used to quantify pathological changes, monitor disease progression, and respond to treatments in patients with muscular dystrophy diseases [23–29]. To date, no studies combining T2 mapping and IDEAL imaging have been conducted on lumbar paraspinal muscles in AS. Thus, our study aimed to assess the lumbar paraspinal muscle microstructural changes in patients with AS compared to matched healthy controls using T2 mapping and IDEAL imaging and to correlate the qMRI results with clinical assessments in AS patients.

## Method

### Patients and healthy controls

A total of 37 patients who underwent radiographic studies of the pelvis to evaluate the sacroiliac joints and MRI examinations in our hospital from December 2019 to November 2020 and met the modified New York criteria for the classification of AS [2] were enrolled in this study. The inclusion criteria were as follows: patients were > 18 years old; had the presence of radiographic sacroiliitis grade 2—4 [3]; had complete clinical information and serological results 3 days prior to the MRI examination. A healthy control group of 37 subjects matched to the AS group in terms of age, body mass index (BMI), and sex distribution was also enrolled to this study (Table 1). Participants were excluded when they presented the following: (1) any neuropsychological disease; (2) injury of back or spine; (3) history of any type of lumbar spine surgery; (4) history of hip fracture surgery and arthroplasty of hip or knee; (5) spinal scoliosis or spondylolisthesis; (6) BMI ≥ 25 kg/m^2^; (7) systematic aerobic or muscular strength training activities; (8) any chronic diseases related to muscle mass loss (such as stroke, parkinsonism, spinal cord injury, cancer, coronary heart disease, thyroid/parathyroid disorder, diabetes mellitus, congestive heart failure, accompanying fibromyalgia, chronic obstructive pulmonary disease, and liver failure); (9) long-term use of corticosteroids due to inflammatory disease; (10) contraindications for MRI (such as cardiac pacemaker, implanted metallic objects, and claustrophobia); and patients who refused to participate in the study. As this trial was a pilot study with non-invasive interventions a trial registry was not performed.

**Table 1:** Demographic and clinical characteristics of AS patients and healthy controls

Variables, N (%) or mean ± SD	AS (*n* = 37)	HC (*n* = 37)	*P-value*
Age, y	27.65 ± 7.32	27.81 ± 3.63	0.610^a^
Sex			
Female	10 (27.1%)	13 (35.1%)	0.451^b^
Male	27 (72.9%)	24 (64.9%)	
BMI, kg/m^2^	20.83 ± 3.21	21.76 ± 2.18	0.149^c^
Current smoking			
Yes	7 (18.9%)	2 (5.4%)	0.152^d^
No	30 (81.1%)	35 (94.6%)	
Alcohol consumption			
Yes	8 (21.6%)	6 (16.2%)	0.553^b^
No	29 (78.4%)	31 (83.8%)	
Exercise habit			
None	21 (56.8%)	16 (43.2%)	0.319^d^
1–2/wk	14 (37.8%)	15 (40.5%)	
≥ 3/wk	2 (5.4%)	6 (16.2%)	
Disease duration, y	6.24 ± 4.56		
ESR^e^, mm/H	22.94 ± 28.31		
CRP^f^, mg/L	20.49 ± 29.33		
BASFI Score (0–10)	0.37 ± 0.70		
BASDAI Score (0–10)	2.46 ± 2.17		
HLA-B27 positive	34 (91.9%)		
TNF inhibitor biological therapy	3 (8.1%)		

*Note*: The values are given as frequencies (percentages) or means ± standard deviation; ^a^ Mann–Whitney *U* test; ^b^ Pearson chi-squared test; ^c^ independent samples *t* test; ^d^ Fisher exact test; A *p*-value of < 0.05 was considered to indicate statistical significance

^e ^Normal range: 0–15 mm/H for female and 0–20 mm/H for male

^f ^Normal range: 0–8 mg/L

*Y* Years, *WK* Week, *ESR* Erythrocyte sedimentation rate, *CRP* C-reactive protein, *BMI* Body mass index, *BASFI* Bath AS function index, *BASDAI* Bath AS disease activity index, *AS* Ankylosing spondylitis, *HC* Healthy control, *HLA-B27* Human leukocyte antigen-B27, *TNF* Tumor necrosis factor

### Demographic and clinical characteristics

Within 3 days of the MRI examination, complete medical histories, physical examinations, and serological tests were available. The demographic variables collated included age, height, weight, alcohol consumption, exercise habit, and current smoking habit. Disease duration, positive HLA-B27 (human leukocyte antigen-B27), erythrocyte sedimentation rate (ESR), C-reactive protein (CRP), and current treatment with tumor necrosis factor (TNF) inhibitors were recorded for the AS group from the patients’ medical reports. The Bath AS Disease Activity Index (BASDAI), which consists of fatigue, spine and peripheral joint pain, sensitivity and morning stiffness measurements [30] and the Bath AS Disease Functional Index (BASFI), which measures the functional capacity of the patient in the previous week [31], were used to assess AS patients. The BASFI index consists of eight questions about daily activities and two questions evaluating the patient’s ability to cope with daily life. The average of the scores assigned by the two rheumatologists was used as the final score. All BASDAI and BASFI scores were measured on a visual analog scale from 0 to 10 and recorded within 1 week of the MRI examination. A BASDAI value of 4 or higher was considered to define active disease. The AS group was then divided into two subgroups: active AS group (BASDAI ≥ 4) and inactive AS group (BASDAI < 4) [32].

### MRI acquisition

Both the AS patients and healthy controls were instructed to avoid physical exercise for 2 days and to sit still for 30 min before the examination. MRI examinations were performed with a 1.5 T MRI unit (EXCITE HDxt, GE Healthcare, USA), according to the protocol used at baseline, in the supine position and feet-first using an 8-channel phased-array abdomen coil. The MRI protocol for lumbar vertebral muscles included localizers, sagittal T1-weighted image, sagittal short-T1 inversion recovery, axial T2 mapping with and without frequency-selective fat saturation (fatsat), and T2 IDEAL sequences. According to information obtained from previous studies, the amount of intramuscular fat significantly increases in the lower lumbar segments for the multifidus (MF) and erector spinae (ES) muscles compared with the upper lumbar segments, and measurement and classification at the L5/S1 level might be inaccurate due to lumbosacral angulation [33, 34]; therefore, only the superior aspect levels of the L3/L4 and L4/L5 intervertebral disks were included in this study. For the axial T2 IDEAL sequence, the following pulse sequences were used: TE, 68 ms; TR, 4850 ms; echo train length, 12; slice thickness, 5 mm; no gap; FOV, 400 × 400 mm; matrix, 256 × 192; NEX, 1; bandwidth, 31.25 kHz; total slices, 10; and acquisition time, 4 min 7 s. Axial T2 mapping sequence with and without fat saturation was acquired (TR, 1000 ms; FOV, 400 × 400 mm; NEX, 1; slice thickness, 5 mm; no gap; matrix, 256 × 192; bandwidth, 31.25 kHz; total slices, 10; and acquisition time, 6 min 28 s) based on multiple echoes (9.7, 19.5, 29.2, 38.9, 48.7, 58.4, 68.2, 77.9 ms).

### MRI analysis

**Fig. 1:**
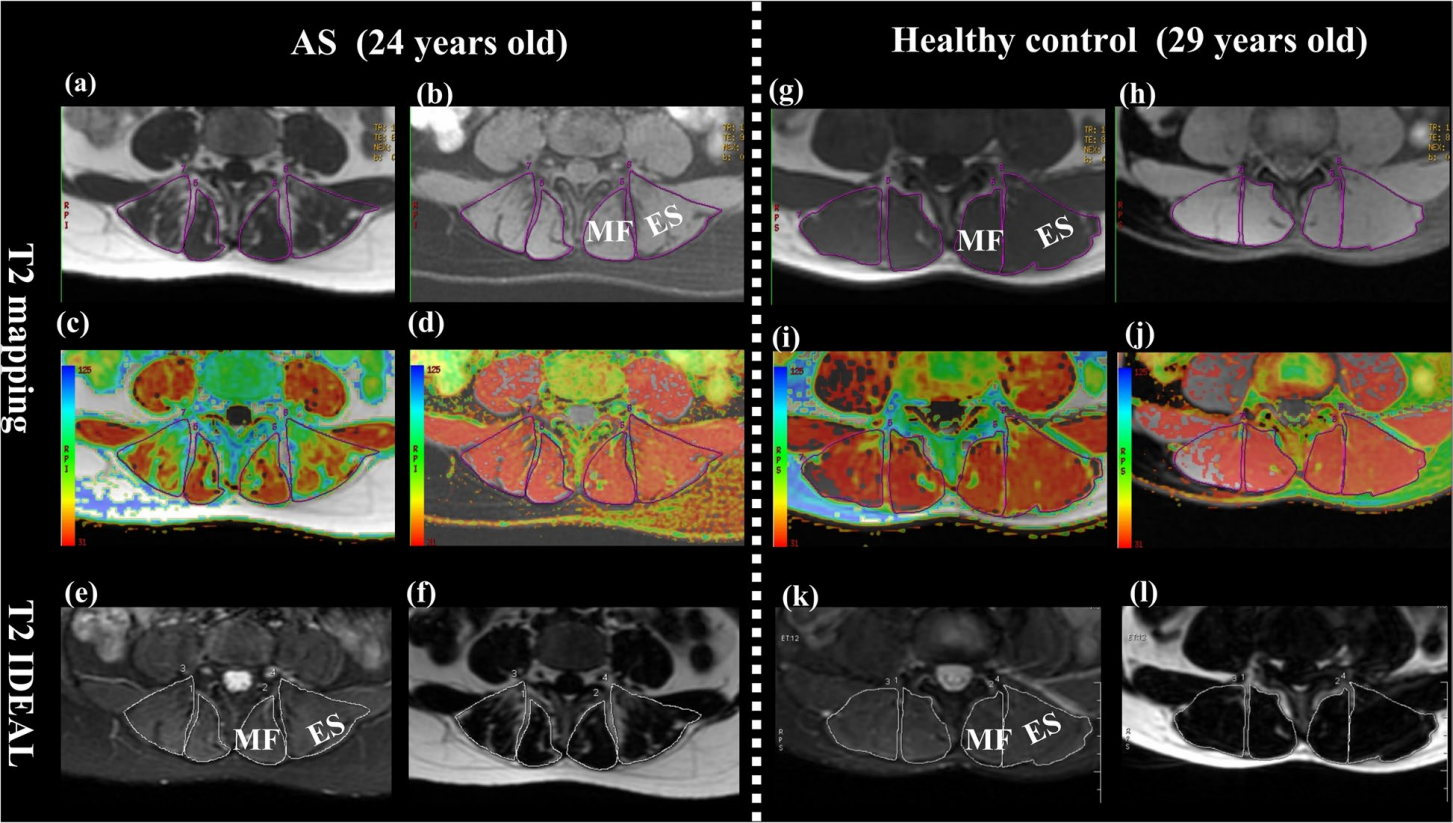
Representative axial T2 and IDEAL maps obtained at the level of L4/L5. Images with manually traced ROIs of multifidus and erector spinae muscles in a 24-year-old patient with AS and a 29-year-old healthy control: (**a**, **g**) conventional T2 mapping without fat saturation; (**b**, **h**) conventional T2 mapping with fat saturation; (**c**, **i**) T2 mapping pseudo-color image without fat saturation; (**d**, **j**) T2 mapping pseudo-color image with fat saturation; (**e**, **k**) T2 IDEAL water image; and (**f**, **l**) T2 IDEAL fat image; note the higher fatty infiltration in the muscles of the patient with AS in comparison to the healthy control. AS, ankylosing spondylitis; IDEAL, iterative decomposition of water and fat with echo asymmetric and least-squares estimation; MF, multifidus; ES, erector spinae

Postprocessing and analysis of the imaging data were conducted on a computer workstation (Advantage Windows Workstation 4.6; GE Healthcare) by two experienced radiologists (H.Y. and R.H. with 3 and 8 years of experience in muscular skeletal radiology, respectively) who were blinded to the study design and the subjects’ clinical information. To measure the inter-examiner reproducibility and the reliability of the MRI quantitative parameters, two radiologists (H.Y. and R.H.) independently performed the measurements twice. There was at least a 2-week interval between each measurement. On the basis of the fat-saturated axial T2WI, regions of interest (ROI) were drawn manually on T2 IDEAL maps and then replicated on the corresponding T2 maps with and without fat saturation in MF and ES at the same level (Fig. 1). ROIs delineated the interior of the muscle and avoided vertebra, fasciae, subcutaneous fat, and blood vessels. The non-fatsat and fatsat T2 relaxation time and cross-sectional area (CSA) on T2 maps were measured separately for the right and left sides, with three consecutive measurements conducted for the right and left MF or ES, which were then averaged. Next, the mean T2_non-fatsat_, T2_fatsat_, and CSA values were obtained by summing the values obtained during the separate measurement on bilateral sides and dividing by two. Finally, the two radiologists' mean value of the measured results was taken as the final result analysis. The FF was calculated using following formula: FF = SI_fat_/(SI_water_ + SI_fat_); whereas the T2_fat_ value was calculated as follows: T2_fat_ value = T2_non-fatsat_ value − T2_fatsat_ value.

### Statistical analysis

SPSS (version 22.0; IBM Corp., Armonk, NY, USA) was used for statistical analyses. Interobserver agreement for the quantitative parameters between two radiologists was assessed using the intraclass correlation coefficient (ICC). An ICC value of 0.61 to 0.80 indicated good agreement, and an ICC value ≥ 0.81 indicated excellent agreement [35]. The normality of each continuous variable was assessed using the Kolmogrov–Smirnov test. The descriptive statistics of the data are presented as mean ± standard deviation (SD) for the continuous data and as frequencies (percentage) for categorical variables. Categorical variables were analyzed using the chi-square test or Fisher’s exact test, and differences in continuous variables were assessed using Student's *t*-test (parametric test) or Mann–Whitney *U* test (non-parametric test). The Spearman’s correlation coefficient model was used to evaluate the correlation between clinical assessments and qMRI results. The relationships were interpreted as strongly, moderately, weakly, and negligibly correlated when *r* ≥ 0.70, *r* = 0.40–0.69, *r* = 0.10–0.39, and *r* ≤ 0.10, respectively [36]. Differences were statistically significant at *p* < 0.05.

## Results

### Subject characteristics

In total, 74 subjects (37 AS patients, 37 healthy controls) completed the study. The demographic and clinical features of the AS patients and healthy controls are presented in Table 1. There were no differences between the groups in terms of age, BMI, current smoking habit, alcohol consumption, exercise habit, or sex distribution (all *p* > 0.05). The clinical features of the AS patients were as follows: the mean ± SD (range) for disease duration and the BASFI and BASDAI scores were 6.24 ± 4.56 years (range 1–21), 0.37 ± 0.70 (range 0–3.8), and 2.46 ± 2.17 (range 0–9.5), respectively. The proportion of patients with active disease (Active AS group) was 21.6%. Radiographic sacroiliitis grade 2 to 4 was detected in 20 (54.1%), 13 (35.1%), and 4 (10.8%) AS cases, respectively. Accelerated ESR and positive CRP were observed in 14 (37.8%) and 15 (40.5%) AS cases, respectively. HLA-B27 was positive in 91.9% of the patients. Of the 37 AS patients, three received current TNF inhibitor therapy.

### Analyses of interobserver agreement

The interobserver agreement of qMRI measurements were evaluated in all patients with the ICCs. As shown in Table 2, SI_fat_, SI_water_, CSA, T2_non-fatsat_, and T2_fastsat_ measures at both L3/L4 and L4/L5 levels showed excellent interobserver agreement (ICC > 0.81).

**Table 2:** Interobserver agreement for MRI quantitative measurements

	L3/L4 level	L4/L5 level
SI_fat_	0.831 (95%CI: 0.717–0.891)	0.914 (95%CI: 0.885–0.949)
SI_water_	0.858 (95%CI: 0.815–0.892)	0.869 (95%CI: 0.817–0.895)
CSA	0.984 (95%CI: 0.979–0.988)	0.961 (95%CI: 0.949–0.971)
T2_non-fatsat_	0.823 (95%CI: 0.761–0.859)	0.926 (95%CI: 0.869–0.965)
T2_fastsat_	0.840 (95%CI: 0.752–0.881)	0.888 (95%CI: 0.854–0.915)

*SI* Signal intensity, *CSA* Cross-sectional area, *MRI* Magnetic resonance imaging, *CI* Confidence interval

### Differences in qMRI measurements between AS group and healthy controls

Compared with the healthy control group, the mean FF and T2_non-fatsat_ values of MF and ES at both L3/L4 and L4/L5 levels were significantly higher, while the mean CSA was significantly lower for the AS group (all *p* < 0.05). The mean T2_fastsat_ values of MF and ES were higher for the AS group than the control group only at the level of L3/L4 (all *p* < 0.05). Although the mean T2_fastsat_ values for MF and ES at the level of L4/L5 were higher in the AS group than the control group, the difference was not statistically significant (all *p* > 0.05). Detailed comparisons of qMRI parameters between the two groups are presented in Table 3.

**Table 3:** Comparison of quantitative MRI measurements between AS patients and healthy controls

Muscle	FF (%)^a^		CSA (mm^2^)^b^		T2_fatsat_ value (ms)^b^		T2_non-fatsat_ value (ms)^a^	
	HC (*n* = 37)	AS (*n* = 37)	HC (*n* = 37)	AS (*n* = 37)	HC (*n* = 37)	AS (*n *= 37)	HC (*n* = 37)	AS (*n* = 37)
L3/4 MF	23.78 ± 4.76	27.98 ± 9.04**	590.48 ± 112.76	526.87 ± 78.81**	37.56 ± 1.72	40.35 ± 3.43***	45.37 ± 3.36	51.05 ± 7.48***
L3/4 ES	25.84 ± 4.98	31.16 ± 7.51***	1838.55 ± 375.75	1575.53 ± 296.72***	37.38 ± 1.94	38.51 ± 2.29*	44.64 ± 4.15	48.62 ± 6.18**
L4/5 MF	27.38 ± 6.01	30.78 ± 7.76*	888.26 ± 93.79	827.01 ± 152.97*	38.77 ± 3.55	39.27 ± 4.52	47.78 ± 5.05	52.50 ± 7.21*
L4/5 ES	30.79 ± 5.45	35.93 ± 7.69***	1459.61 ± 278.13	1276.23 ± 237.94**	38.17 ± 6.24	39.50 ± 2.52	47.68 ± 5.08	52.73 ± 7.51***

*Note*: The values are given as means ± standard deviation; ^a^ independent samples *t* test; ^b^ Mann–Whitney *U* test

*AS* Ankylosing spondylitis, *HC* Healthy control, *MF* multifidus, *ES* erector spinae, *FF* Fat fraction, *CSA* Cross-sectional area

^*^P < 0.05, **P < 0.01, ***P < 0.001, versus healthy controls

### Correlation between qMRI and clinical assessments in AS patients

There was no statistical correlation between the qMRI measurements and BASDI and CRP (all *p* > 0.05). In terms of FF measurements, the mean FF of the MF and ES at both the L3/L4 and L4/L5 levels were weakly to moderately correlated with disease duration (*r* = 0.318–0.359, all *p* < 0.05) and age (*r* = 0.362–0.415, all *p* < 0.05) respectively. Age and disease duration were determined to have a significant positive correlation (*r* = 0.528, *p* < 0.001). Since increased fat deposition in paraspinal muscles has been studied throughout aging [37], partial correlation testing was then performed with age as a control variable for FF and disease duration, and no significant link was found in both MF and ES at all levels (all *p* > 0.05). A weak to moderate negative correlation was obtained between the mean CSA in MF at both L3/L4 and L4/L5 levels and ESR (*r* =  − 0.503 and − 0.409, all *p* < 0.05, respectively).

Regarding the T2 mapping measurements, there were weak to moderate statistical positive correlations between the mean T2 values with and without fatsat and ESR in MF and ES at both L3/L4 and L4/L5 levels (Fatsat, *r* = 0.333–0.426, all *p* < 0.05; Non-fatsat, *r* = 0.385–0.599, all *p* < 0.05, respectively). In addition, a weak positive relationship was found for T2 values with and without fatsat in MF and ES only at the level of L3/L4 with BASFI (Fatsat, *r* = 0.410 and 0.379, all *p* < 0.05; Non-fatsat, *r* = 0.371 and 0.356, all *p* < 0.05, respectively). The results of detailed Spearman’s analysis of correlations between clinical assessments and qMRI results are presented in Table 4.

**Table 4:** Correlation between quantitative MRI and clinical assessments in AS group

		Disease duration^a^	ESR	CRP	BASFI	BASDAI	Age
L3/4 MF	FF	0.129	0.274	0.061	0.130	-0.145	0.415*
	T2_non-fatsat_ value	0.281	0.599**	0.319	0.371*	0.064	0.220
	T2_fatsat_ value	-0.134	0.419**	0.307	0.410*	0.281	-0.067
	CSA	0.059	-0.503**	-0.114	-0.157	0.019	-0.070
L3/4 ES	FF	0.118	0.293	0.059	0.077	-0.125	0.362*
	T2_non-fatsat_ value	0.149	0.511**	0.220	0.356*	-0.025	0.168
	T2_fatsat_ value	-0.293	0.426**	0.167	0.379*	0.236	-0.267
	CSA	0.228	-0.216	-0.006	-0.261	0.021	0.260
L4/5 MF	FF	0.147	0.232	0.055	0.186	-0.030	0.394*
	T2_non-fatsat_ value	0.224	0.503**	0.257	0.227	0.082	0.239
	T2_fatsat_ value	0.152	0.418*	0.189	0.067	0.090	0.063
	CSA	0.054	-0.409*	-0.047	-0.152	0.215	0.135
L4/5 ES	FF	0.153	0.286	0.092	0.111	-0.065	0.395*
	T2_non-fatsat_ value	0.038	0.385*	0.098	0.217	0.124	0.221
	T2_fatsat_ value	-0.323	0.333*	-0.021	0.170	0.134	-0.060
	CSA	0.305	-0.210	-0.018	-0.109	-0.060	0.232

*Note*: *r*, Spearman’s correlation test coefficient; *, *P* < 0.05; **, *P* < 0.01

*ESR* Erythrocyte sedimentation rate, *CRP* C-reactive protein, *BASDAI* Bath AS disease activity index, *FF* Fat fraction, *MF* Multifidus, *ES* Erector spinae, *CSA* cross-sectional area

^a^, partial correlation testing adjusting for age

### Associations between CSA, FF, and T2 values in AS patients

**Fig. 2:**
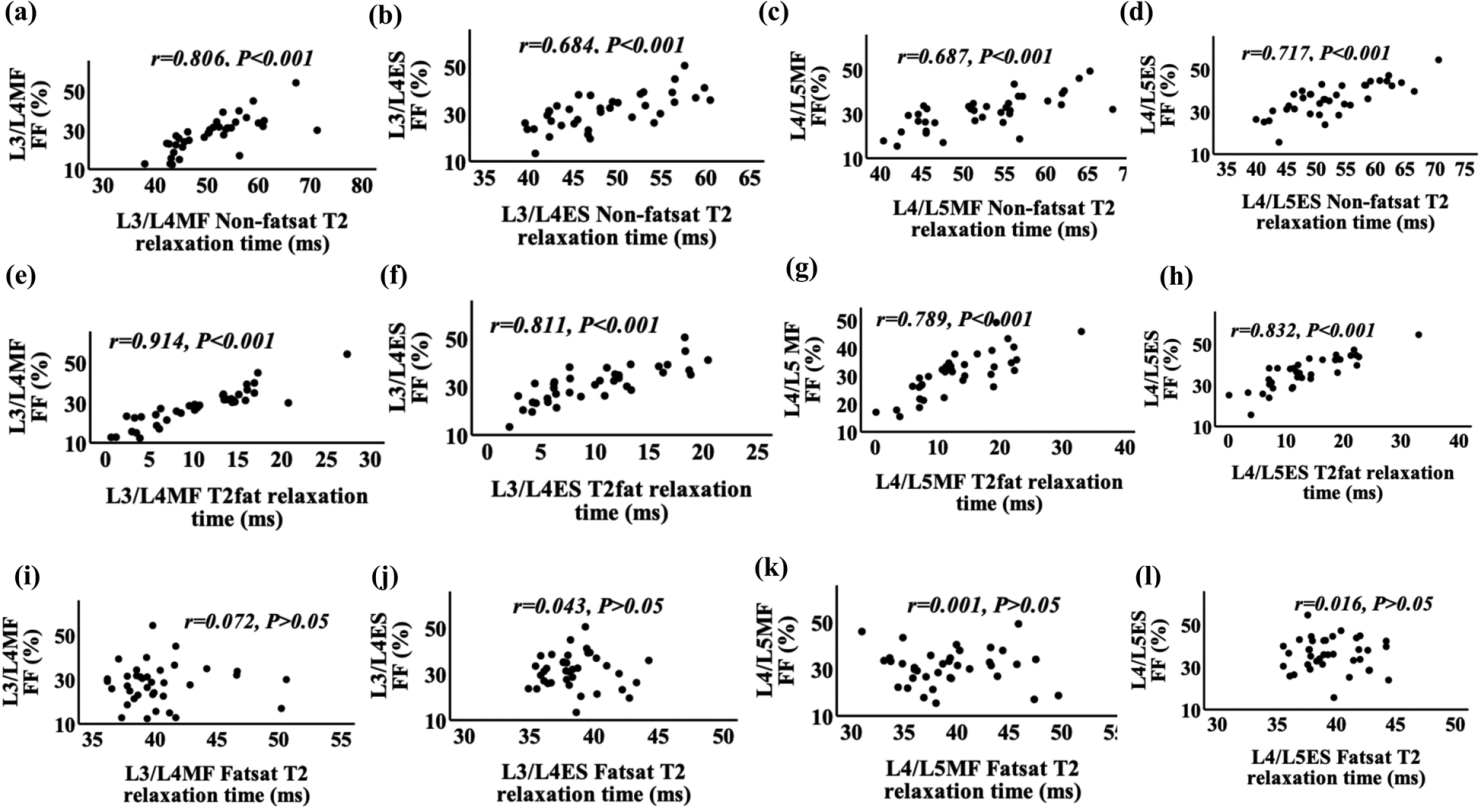
Scatter plots showing correlation of FF and the T2_non-fatsat_ value (**a-d**) and the T2_fat_ value (**e–h**) and T2_fatsat_ value (**i-l**) in ES and MF at both L3/L4 and L4/L5 levels in AS group. In the upper row (**a-d**), scatter plots showing a moderately to strongly positive correlations between FF and the T2_non-fatsat_ value in MF and ES at all levels. In the middle row (**e–h**), scatter plots showing strongly positive correlations between FF and the T2_fat_ value in MF and ES at all levels. In the lower row (**i-l**), no significant correlation were observed between FF and the T2_fatsat_ value in MF and ES at all levels. AS, ankylosing spondylitis; MF, multifidus; ES, erector spinae; FF, fat fraction

There was a statistically significant and moderately to strongly positive correlation between the mean FF and T2_non-fatsat_ value (Fig. 2a-d) or T2_fat_ value (Fig. 2e-h) for all muscles (all *p* < 0.001); however, no correlation was found between the mean FF and T2_fatsat_ value in any muscles (Fig. 2i-l). When we compared the mean CSA with the FF or T2_non-fatsat_ value, a significant weak negative correlation was found only for MF at the level of L3/L4 (*r* =  − 0.328 and *r* =  − 0.383, all *p* < 0.05) (Supp. Table 1). There was a weakly negative correlation between the mean CSA and T2_fatsat_ value in MF and ES at the levels of L3/L4 (*r* =  − 0.433 and − 0.361, all *p* < 0.05). The detailed correlation results for qMRI measurements in AS patients are shown in Fig. 2 and Supplementary Table 1.

### Comparison of clinical and qMRI variables between active and inactive AS groups

A comparison of the clinical and qMRI results for the active and inactive AS groups (Table 5) showed that the active AS group had a significantly shorter disease duration; higher ESR, CRP, and BASDAI scores; and higher T2_fatsat_ values for MF and ES at the level of L3/L4 (all *p* < 0.05). No differences were observed between the two groups in terms of age, sex, BMI, BASFI, CSA, FF, or T2_non-fatsat_ values for MF and ES at either the L3/L4 or L4/L5 levels, nor for the T2_fatsat_ value for MF and ES at the level of L4/L5 (all *p* > 0.05).

**Table 5:** Comparison of clinical and quantitative MRI data between active and inactive AS groups

Variables, N (%) or mean ± SD	Active AS group (*n* = 8)	Inactive AS group (*n* = 29)	*P value*
Clinical data			
Age, y	26.38 ± 3.82	28.00 ± 8.05	0.899^**a**^
Sex/M	6 (75%)	21 (72.4%)	1.000^**b**^
BMI, kg/m^2^	20.41 ± 3.78	20.94 ± 3.11	0.731^**c**^
Disease duration, y	3.37 ± 2.51	7.03 ± 4.71	**0.021**^**a**^
ESR, mm/H	45.75 ± 46.79	16.65 ± 17.12	**0.008**^**a**^
CRP, mg/L	42.24 ± 46.08	14.50 ± 20.07	**0.016**^**a**^
BASFI Score (0–10)	0.91 ± 1.27	0.22 ± 0.35	0.118^**a**^
BASDAI Score (0–10)	5.91 ± 1.59	1.52 ± 1.06	**0.000**^**a**^
Quantitative MRI assessments			
L3/4 MF CSA, mm^2^	534.38 ± 69.42	524.81 ± 82.21	0.766^**a**^
L3/4 ES CSA, mm^2^	1619.63 ± 396.71	1563.36 ± 270.45	0.642^**a**^
L4/5 MF CSA, mm^2^	863.75 ± 122.51	816.87 ± 160.76	0.451^**a**^
L4/5 ES CSA, mm^2^	1288.00 ± 240.29	1272.98 ± 241.47	0.877^**a**^
L3/4 MF FF, %	27.32 ± 8.63	28.16 ± 9.29	0.871^**c**^
L3/4 ES FF, %	30.38 ± 9.26	31.38 ± 7.13	0.814^**c**^
L4/5 MF FF, %	32.07 ± 9.05	30.43 ± 7.51	0.299^**c**^
L4/5 ES FF, %	35.70 ± 9.97	35.99 ± 7.16	0.651^**c**^
L3/4 MF T2_non-fatsat_ value, ms	54.69 ± 9.64	50.46 ± 6.64	0.124^**c**^
L3/4 ES T2_non-fatsat_ value, ms	50.98 ± 6.84	47.94 ± 5.94	0.226^**c**^
L4/5 MF T2_non-fatsat_ value, ms	55.91 ± 8.96	51.56 ± 6.52	0.132^**c**^
L4/5 ES T2_non-fatsat_ value, ms	56.52 ± 7.86	51.68 ± 7.20	0.108^**c**^
L3/4 MF T2_fatsat_ value, ms	42.74 ± 4.75	39.69 ± 2.72	**0.024**^**a**^
L3/4 ES T2_fatsat_ value, ms	40.09 ± 2.62	38.07 ± 2.03	**0.026**^**a**^
L4/5 MF T2_fatsat_ value, ms	40.11 ± 4.89	39.04 ± 4.48	0.559^**a**^
L4/5 ES T2_fatsat_ value, ms	40.73 ± 2.68	39.17 ± 2.41	0.123^**a**^

*Note*: The values are given as frequencies (percentages) or means ± standard deviation; ^a^ Mann–Whitney *U* test; ^b^ Pearson chi-squared test; ^c^ independent samples *t* test; A *p*-value of < 0.05 was considered to indicate statistical significance

*Y* years, *M* male, *AS* Ankylosing spondylitis, *ESR* Erythrocyte sedimentation rate, *CRP* C-reactive protein, *BMI* body mass index, *BASFI* Bath AS function index, *BASDAI* Bath AS disease activity index, *FF* Fat fraction, *MF* Multifidus, *ES* Erector spinae, *CSA* Cross-sectional area

## Discussion

In this study, we used T2 mapping and IDEAL to compare changes in the composition of paraspinal muscles between AS patients and matched healthy controls. Correlations between qMRI results and clinical assessments were analyzed in AS patients. In addition, we also compared the clinical and qMRI variables of patients with active and inactive AS. Our main findings were as follows: (i) T2_non-fatsat_ values and FF were elevated and CSA was decreased in MF and ES at both L3/L4 and L4/L5 levels in the AS group compared to the healthy controls; (ii) T2_fatsat_ values were higher in MF and ES at the L3/L4 level in AS compared to controls, and they were also significantly higher in the active AS compared to the inactive AS group.

Studies revealed that the evaluation of fatty infiltration in lower lumbar segments can be best represented that of the entire lumbar region and could reflect the morbid state of patients with low back pain [34, 38]. In addition, an inaccuracy of measurement and classification can occur at the L5/S1 level due to lumbosacral angulation [33]. Thus, the assessment of ES and MF has been performed at both the L3/L4 and L4/L5 levels. In the current study, FF and CSA in MF and ES at both L3/L4 and L4/L5 levels in AS patients were significantly higher than those in the matched healthy control group. These findings are in accordance with those of previous pathological and morphological MRI studies [14, 18, 19]. In their cohort, Akgul et al. reported that fatty infiltration of the paraspinal muscle was a feature of both established AS and non-radiological axial spondyloarthritis, but it was more pronounced in the former [18]. In another study conducted by Resorlu et al., lumbar paraspinal muscles of AS patients and healthy controls were evaluated by conventional MRI with a visual semi-quantitative method, and fatty infiltration and muscular atrophy were observed more often in the AS group at almost all levels [19]. Recently, Bok et al. demonstrated a significantly lower volume of paraspinal muscle in early-stage AS patients without spinal deformities compared to chronic back pain patients [39]. The exact mechanisms responsible for the decrease in muscle volume and increase in fatty infiltration in AS patients are not well understood. However, several theories have been proposed, including (1) systemic inflammation causing the overexpression of TNF-α and other proinflammatory cytokines (interleukins 6, 1, 17, and 23), which have been reported to be involved in myofibril protein catabolism; (2) local inflammation at the site of attachment of ligaments or tendons to bone (enthesitis) or facet joint arthritis; (3) biomechanical stress; and (4) denervation or/and neuroinflammation of the spinal cord and nerve roots [8, 14, 16, 20–22, 40–43]. Other factors that have a bidirectional role (i.e., can be both a cause and consequence of vicious cycle initiation) of paraspinal muscle degeneration in AS include lower back pain, facet joint or spinal stiffness, inactivity, and fatigue [9, 11, 32, 33, 39, 44]. In addition, recent studies indicate that MF adipose tissue is also a source of TNF-α and interleukin 6 expression [22, 45]. Nevertheless, none of these mechanisms alone can explain the phenomenon; therefore, various combinations of these factors may be related to muscular degeneration pathogenesis during the disease progress.

It has been reported that T2 relaxation times depend strongly on the water content of tissue [27]. However, an elevated T2 relaxation time can also be influenced by fat deposition within skeletal muscle tissue in neuromuscular disorders [27–29]. According to previous histopathological studies of paraspinal muscles in AS, different severities and extents of inflammatory cell infiltration, muscular edema, fatty infiltration, and fibrosis, which may potentially affect the T2 value, have been observed according to differences in the disease duration and severity of AS [20–22]. It is known that edema/inflammation and fat infiltration have longer T2 values, while fibrosis lies on the opposite side of the T2 spectrum (i.e., a shorter mean T2 value) [24, 26]. In this study, the mean T2_non-fatsat_ values of the paraspinal muscle at both the L3/L4 and L4/L5 levels were remarkably elevated in AS patients compared to that of matched healthy controls. These results are in concordance with data from a previous study by Otto et al. [23]. Zhai et al. recently reported that the presence of chronic fibrosis in patients with inactive stage of thyroid-associated ophthalmopathy might adversely influence the FF value in muscles using T2 IDEAL technique [46]. In the current study, we determined the existence of strong correlations between FF and the T2_non-fasat_ and T2_fat_ values in all paraspinal muscles, indicating the predominant microscopic finding was fatty infiltration in paraspinal muscles in AS. These findings show that it may not be adequate to assess the degenerative changes to paraspinal muscles in AS only using T2 mapping without fat saturation or T2 IDEAL.

Interestingly, after fat saturation, the T2_fatsat_ value was still significantly higher in ES and MF at the level of L3/L4 but not at L4/L5 in AS patients compared to healthy controls; likewise, a significantly increased T2_fatsat_ value in ES and MF at the level of L3/L4 was also observed in the active AS group compared to the inactive AS group. These findings are similar to those of a study conducted by Arpan et al., who demonstrated that patients with Duchenne muscular dystrophy, even young children, had muscles with elevated mean T2, were more heterogeneous, and had a greater percentage of elevated pixels in their lower leg muscles than controls [27]. T2 measurements decreased with fat saturation, but were still higher (*p* < 0.05) in dystrophic muscles than controls [27]. As confirmed by previous pathological studies, the myopathic processes in paraspinal muscles of AS patients are patchy and heterogeneous, and hence, lead to different degrees of inflammatory cell infiltration [20–22, 47, 48]. The inflammatory state may increase sarcolemma permeability and decrease adenosine triphosphate levels, causing dysfunction of sodium/potassium pumps with a consequent shift of water from the interstitial to the intracellular space, subsequently resulting in increased intracellular free water with increasing T2 relaxation times of muscle tissue [28, 29]. Therefore, we think that the elevated T2_fatsat_ in AS subjects might be related to the inflammation and edema associated with muscle damage. Further studies on the relationship between inflammatory cell infiltration/inflammatory muscular edema and the T2_fatsat_ value are necessary.

In the present study, FF was significantly correlated with age and disease duration in AS patients. Our results were in agreement with the findings of a previous study [19]. Resorlu et al. revealed a positive correlation between the duration of disease and fatty infiltration in paraspinal muscles at four levels between L1 and L5 in patients with AS [19]. However, no significant positive correlation was found between FF and disease duration after adjusting for age in our study. This seemingly contradictory finding may be explained by the fact that our analysis comprised a relatively young study population (27.65 ± 7.32 years old) with shorter disease duration (6.24 ± 4.56 years) as compared to the AS patients (mean age: 42.57 ± 11.45 years old; disease duration: 11.05 ± 9.20) included by Resorlu et al. These data suggest that the degree of paraspinal muscle fatty infiltration in young AS patients during the early stage may be related to inflammation, pain, stiffness, and entheses, other than disease duration and structural deformity. ESR, CRP, BASFI, and BASDAI exhibited no significant correlation with FF. In contrast, to our surprise, weak to moderate positive correlations between ESR and T2 values with and without fat saturation were observed in AS patients. Possible explanations for these conflicting results are that BASFI and BASDAI are fully patient-reported and, therefore, reflect subjective factors independent of inflammation. In addition, the ESR is unspecific and may be more easily affected by different conditions with longer half-value periods, even in subacute/chronic inflammation, compared to CRP [11, 15, 31]. Nonetheless, more studies are needed to understand the reasons for these results.

In contrast to previous studies, our study can be considered to be strong in the following aspects. This was the first study that used a combination of T2 mapping with and without fat saturation and IDEAL to assess paraspinal muscle degeneration in AS. We controlled for paraspinal muscle degeneration by matching AS patients and healthy controls not only for age, sex, and BMI but also for personal factors such as current smoking, alcohol consumption, and exercise habits, which have the potential to influence microstructure changes in paraspinal muscle.

The present study is not without limitations. First, the sample size was not very large; thus, further studies involving patients with various categories of disease severity and duration are needed. Second, the ROIs were placed only within a single slice and may not have been totally representative of the parameters assessed throughout the muscles of the lower back. Third, although there were no associations between disease activity measurements (CRP and BASDAI) and FF or T2 values in AS patients, we cannot rule out the possibility that the low proportion of active AS patients (21.6%) in this study affected the results. In addition, because this study was cross-sectional, we could not test for causal relationships between variables.

## Conclusions

In conclusion, our findings demonstrated the presence of decreased CSA and increased FF and T2_non-fatsat_ values for lumbar paraspinal muscles in AS patients. The elevated T2_fatsat_ values in AS patients, especially in the active stage, may be related to inflammatory cell infiltration/muscular edema. These results indicate that using a combination of IDEAL and T2 mapping may provide useful global information and deeper insights into the pathophysiological degeneration of paraspinal muscles in AS. Future longitudinal studies with qMRI examinations of paraspinal muscles to extend the findings of the present study will be valuable.

## Supplementary Information

**Additional file 1:** **Supplementary Table 1.** Correlation between CSA, FF, and T2 relaxation time in AS.

## Data Availability

Datasets used and/or analyzed in the current study are available from the corresponding author on reasonable request.

## Abbreviations

AS: Ankylosing spondylitis
ESR: Erythrocyte sedimentation rate
CRP: C-reactive protein
BMI: Body mass index
BASFI: Bath AS Function Index
BASDAI: Bath AS disease activity index
HLA-B27: Human leukocyte antigen-B27
TNF: Tumor necrosis factor
MF: Multifidus
ES: Erector spinae
FF: Fat fraction
CSA: Cross sectional area
IEDAL: Iterative decomposition of water and fat with echo asymmetric and least-squares estimation
QMRI: Quantitative magnetic resonance imaging
