# Supplementary material for: T2 mapping and fat quantification of lumbar paraspinal muscle in ankylosing spondylitis: a case control study
Source: BMC Musculoskelet Disord. 2022 Jun 27;23:614. doi: 10.1186/s12891-022-05570-9 (PMC9235229; doi:10.1186/s12891-022-05570-9)
Supplement: Supplementary file 1 — Additional file 1: Supplementary Table 1. Correlation between CSA, FF, and T2 relaxation time in AS. [file 12891_2022_5570_MOESM1_ESM.docx]

| **Supplementary Table 1** Correlation between CSA, FF, and T2 relaxation time in AS | | | | | |
| --- | --- | --- | --- | --- | --- |
|  |  | L3/4MF CSA | L3/4ES CSA | L4/5MF CSA | L4/5ES CSA |
| L3/4MF | FF | **-0.328*** | - | - | - |
|  | T2_non-fatsat_ value | **-0.383*** | - | - | - |
|  | T2_fatsat_ value | **-0.433**** | - | - | - |
|  | T2_fat_ value | -0.231 | - | - | - |
| L3/4ES | FF | - | -0.152 | - | - |
|  | T2_non-fatsat_ value | - | -0.266 | - | - |
|  | T2_fatsat_ value | - | **-0.361*** | - | - |
|  | T2_fat_ value | - | -0.137 | - | - |
| L4/5MF | FF | - | - | -0.037 | - |
|  | T2_non-fatsat_ value | - | - | -0.141 | - |
|  | T2_fatsat_ value | - | - | -0.105 | - |
|  | T2_fat_ value | - | - | 0.110 | - |
| L4/5ES | FF | - | - | - | 0.093 |
|  | T2_non-fatsat_ value | - | - | - | -0.111 |
|  | T2_fatsat_ value | - | - | - | 0.038 |
|  | T2_fat_ value | - | - | - | -0.301 |

Note: *r,* Spearman’s correlation test coefficient; *****, *P* < 0.05; ******, *P* < 0.01

Abbreviations: AS, ankylosing spondylitis; FF, fat fraction; MF, multifidus; ES, erector spinae; CSA, cross-sectional area.
